# Association of cardiovascular events and lipoprotein particle size: Development of a risk score based on functional data analysis

**DOI:** 10.1371/journal.pone.0213172

**Published:** 2019-03-07

**Authors:** Charles M. Rowland, Dov Shiffman, Michael Caulfield, Veronica Garcia, Olle Melander, Trevor Hastie

**Affiliations:** 1 Quest Diagnostics, San Juan Capistrano, CA, United States of America; 2 Department of Clinical Sciences, Lund University, Malmö, Sweden; 3 Department of Statistics, Stanford University, Stanford, CA, United States of America; International University of Health and Welfare, School of Medicine, JAPAN

## Abstract

**Background:**

Functional data is data represented by functions (curves or surfaces of a low-dimensional index). Functional data often arise when measurements are collected over time or across locations. In the field of medicine, plasma lipoprotein particles can be quantified according to particle diameter by ion mobility.

**Goal:**

We wanted to evaluate the utility of functional analysis for assessing the association of plasma lipoprotein size distribution with cardiovascular disease after adjustment for established risk factors including standard lipids.

**Methods:**

We developed a model to predict risk of cardiovascular disease among participants in a case-cohort study of the Malmö Prevention Project. We used a linear model with 311 coefficients, corresponding to measures of lipoprotein mass at each of 311 diameters, and assumed these coefficients varied smoothly along the diameter index. The smooth function was represented as an expansion of natural cubic splines where the smoothness parameter was chosen by assessment of a series of nested splines. Cox proportional hazards models of time to a first cardiovascular disease event were used to estimate the smooth coefficient function among a training set consisting of one half of the participants. The resulting model was used to calculate a functional risk score for the remaining half of the participants (test set) and its association with events was assessed in Cox models that adjusted for traditional cardiovascular risk factors.

**Results:**

In the test set, participants with a functional risk score in the highest quartile were found to be at increased risk of cardiovascular events compared with the lowest quartile (Hazard ratio = 1.34; 95% Confidence Interval: 1.05 to 1.70) after adjustment for established risk factors.

**Conclusion:**

In an independent test set of Malmö Prevention Project participants, the functional risk score was found to be associated with cardiovascular events after adjustment for traditional risk factors including standard lipids.

## Introduction

Functional data can be represented by functions that describe curves or surfaces of a low-dimensional index[1]. Examples of functional data are measurements collected over time, across locations, or, as in the present analysis, across a vector of particle diameters. Functional data analysis exploits these data attributes by imposing structure: for example, smoothness on regression coefficients, as a function of the same index[2]. Regression modelling of functional data has been, and continues to be, an active area of methodological development and has been used in a variety of applications[3, 4]. A previous publication provided several examples of functional data analysis within the field of medical science in order to increase awareness of the challenges and possibilities involved, and to encourage scientists to explore the robustness of functional approaches in additional applications[5]. While some aspects of functional data analysis may be common across a wide range of applications, it is likely that each application will also have unique challenges worthy of exploration. The current paper explores a functional data analysis approach to assess the association between size-fractionated lipoprotein particles and cardiovascular disease (CVD).

CVD is the most common cause of morbidity and mortality in the US[6]. Established risk factors for CVD—such as hypertension, high levels of low-density-lipoprotein cholesterol (LDL-C), low levels of high-density-lipoprotein cholesterol (HDL-C), smoking, male gender, and old age—do not entirely account for CVD risk [7]. Since treating modifiable risk factors is known to reduce the risk of CVD[8, 9], improving CVD risk stratification would enable a better allocation of prevention resources [10]. And one approach to improving risk prediction is to consider risk of CVD associated with the size distribution of a patient’s lipoprotein particles.

Lipoproteins comprise proteins and lipids (e.g., cholesterol and phospholipids) assembled into particles with a hydrophilic exterior and hydrophobic interior. These particles transport hydrophobic lipids through the circulatory system. Depending on their composition, lipoproteins vary in both size (from 7nm to several hundred nm) and density[11]. Although lipoproteins have a continuous size distribution, they are typically categorized as high density (HDL), low density (LDL), intermediate density (IDL), and very low density (VLDL) lipoproteins; HDL are the smallest and VLDL are the largest. These classes have been further characterized into finer size and/or density subclasses[12]. Clinical studies have provided evidence that patients whose total lipoprotein particle burden is comprised of a relatively greater proportion of smaller, denser LDL particles may be at increased risk compared with patients whose burden is comprised mostly of larger, less dense LDL particles[13].

The size or density of lipoprotein particles can be determined by a variety of methods, including ion mobility[14], nuclear magnetic resonance [15], and ultracentrifugation [16, 17]. Analysis of ion mobility (a type of gas phase electrophoresis) data typically proceeds by first combining the lipoprotein particle abundance measures into predetermined regions defined by particle diameter (e.g. LDL-small, LDL-medium, LDL-large) [18]. However, the diameter boundaries used to define these regions are somewhat arbitrary, and information about risk of CVD could potentially be lost or diminished if, for example, a defined region contained a mixture of risk-increasing and risk-decreasing species of particles.

The data produced by ion mobility analysis for a particular sample are a vector of particle abundance measures associated with a corresponding (one to one) vector of particle size diameters of a range known to include HDL, LDL, IDL, and VLDL particles. To avoid the arbitrary nature of defining size regions, it may be preferable to use the complete vector of size specific particle abundance measures of each sample in the analysis. Since abundance values of particles with similar diameters are highly correlated, it is reasonable to assume that the risk of CVD would vary as a smooth function of the abundance measures. The data generated from ion mobility analysis can be considered functional data and functional data analysis could be a useful approach to model the association of such data with CVD.

In this paper, we use functional data analysis to model the association of the ion mobility data and traditional risk factors with CVD. The advantages of this approach include summarizing this association with a single patient functional risk score and graphing the smoothed regression coefficients according to particle diameter to help identify the most informative size regions. First, the methods and general functional approach to modeling the ion mobility data are described. Next, the results applying the approach to subjects collected by the Malmö Prevention Project (MPP) study are summarized. Finally, the type I error and power of the approach are evaluated through simulations.

## Materials and methods

### Description of data

For each of *N* subjects, the data consists of CVD event status, time to event or last follow-up, a vector of lipoprotein particle abundance at each of *J* particle diameters, and possibly other covariates including demographic characteristics and traditional risk factors for CVD such as HDL-C and LDL-C. Particle abundance, measured at each diameter value, can be recorded as particle number concentration (number/mL), particle mass concentration (mass/mL), or as in the applied example, an arbitrary mass unit using the formula: *mass* = *density* × *volume* (assuming a density of 1.0 for all lipoprotein particles)[14].

### Creating the functional model

Suppose for subject *i*, ***x***_*i*_ is a vector of particle mass, with ***x***_*ij*_ being the mass at size *s*_*j*_, and *j* running from 1,⋯,*J*. While there are many ways one could reduce this variable to a single score or number, we choose to use a linear functional:

$$f(x_{j})=\sum_{j=1}^{J}\beta (s_{j})x_{ij}=\sum_{j=1}^{J}\beta_{j}x_{ij}$$

Here, *β*(*s*) is a coefficient function, a function of particle size (*s*). Since one might expect the contributions of particle mass for neighboring sizes to be similar, it is reasonable to assume *β*(*s*) is smooth as a function of *s*. We can achieve this by representing *β*(*s*) in a basis of smooth functions, such as natural cubic splines of order *M* with suitably placed knots:

$$\beta (s)=\sum_{m=1}^{M}h_{m}(s)\theta_{m}$$

An advantage of the linear functional is that it can be incorporated within standard regression models, such as a Cox model. For the current example, we wish to model:

$$\log\;\lambda_{it}=\alpha_{t}+x_{i}\prime \beta +z_{i}\prime \gamma$$

where *λ*_*it*_ represents the hazard of a CVD event for subject *i* at time *t*, *α*_*t*_ is a baseline hazard at time *t*, ***x***_*i*_ is the vector of particle mass and ***z***_*i*_ a vector of *r* covariates for the *i*th subject, ***γ*** is an *r* by 1 vector of regression coefficients corresponding to the covariates, and ***β*** is defined below.

With our above definition of *β*(*s*), we can now write the vector ***β*** = (*β*(*s*_1_),*β*(*s*_2_),⋯,*β*(*s*_*J*_))′ as: ***β*** = ***Hθ*** where ***H*** is the *J* by *M* matrix of basis vectors with ***H***_*jm*_ = *h*_*m*_(*s*_*j*_), and ***θ*** an *M* by 1 vector of regression coefficients. In subsequent text, we refer to ***H*** as a “filter”.

Substituting back in the original model,

$$\log\;\lambda_{it}=\alpha_{t}+x_{i}\prime (H\theta )+z_{i}\prime \gamma$$

However, we can use the filter ***H*** on ***x*** first,

$$\log\;\lambda_{it}=\alpha_{t}+(x_{i}\prime H)\theta +z_{i}\gamma$$

Thus, we obtain ***XH***, a matrix with *N* rows and *M* columns, that can be entered as *M* variables in a Cox regression model in order to estimate ***θ***.

Since the estimated reduced set of coefficients, $\hat{\theta }$, are not easily interpretable, we can subsequently back transform using ***H*** to get our desired estimate:

$$\hat{\beta }(s)=\sum_{m=1}^{M}h_{m}(s){\hat{\theta }}_{m}$$

A likelihood ratio test with *M* degrees of freedom is performed to test whether the particle mass data, represented by ***XH***, results in a significant improvement when compared with a model having only covariates ***Zγ*.** A covariance matrix, $\hat{\Sigma }$, for ***β***, is calculated from the covariance matrix of $\hat{\theta }$ (determined from the Cox model) and the filter ***H***:

$$\hat{\Sigma }=H\left(cov(\hat{\theta })\right)H^{\prime }$$

The variance for $\hat{\beta }(s)$ at any *s* is: $var\left(\hat{\beta }(s)\right)=h{\left(s\right)}^{\prime }cov(\hat{\theta })h(s)$ where ***h***(*s*) = (*h*_1_(*s*),*h*_2_(*s*),⋯,*h*_*m*_(*s*))′

Approximate 95% confidence intervals are then obtained as:

$$\hat{\beta }(s)\pm 2\sqrt{var(\hat{\beta }(s))}$$

$\hat{\beta }(s)$ and corresponding 95% confidence intervals can be plotted versus size (*s*), highlighting regions of the profile where the confidence interval does not contain zero.

### Selecting the degrees of freedom

We use a basis of natural cubic splines with knots chosen at uniformly spaced values of *s*. For natural splines with *M* knots, one gets *M* basis functions, and hence the "degrees of freedom" are *M*.

It is unknown how smooth the function needs to be to result in good prediction of the outcome. We use a “nested spline” procedure that allows only a small number of choices for smoothness. Specifically, we evaluate a series of degree of freedom (*df*) choices that result in nested regression models and apply likelihood ratio tests to determine the best choice of *df* from the series. A nested series of splines occurs when the knots of a more complex spline include the identical knots of a less complex spline with additional knots that further subdivide the space (S1 Fig). For natural cubic splines with an intercept, and *k* + 1 being the lowest number of *df* desired, a series of *df* choices resulting in *q* nested models was obtained by the equation:

$${df}_{q}=k(2^{j-1})+1\;\mathrm{for}\;j\;\mathrm{in}\;1,\;2,\ldots ,q$$

After simulating a variety of potential functional effects, we determined that degrees of freedom in the range of 7 to 16 typically provided a reasonable fit to the function (S2 Fig) and thus explore two possible series: “7 and 13” and “8 and 15”. In the case of two choices for *M*, the less complex of the two models is selected unless a likelihood ratio test comparing the two models is significant. The chosen model is then compared to a null model (or a model having only covariates) while adjusting for multiple testing using standard methods (e.g. Bonferroni). Similar methods could be devised if more than 2 choices for *M* are desired.

### Calculation of functional risk scores for new subjects

Functional risk scores for new subjects are determined as ${score}_{i}=(x_{i}\prime H)\hat{\theta }+z_{i}\prime \hat{\gamma }$ if covariates are available on the new subjects, or as ${score}_{i}=(x_{i}\prime H)\hat{\theta }$

In the current paper, the latter equation with $\hat{\theta }$ representing the *M* regression coefficients from the Cox model fit to the training set was used to calculate functional risk scores for test set subjects in order to assess the effect of the ion mobility data (represented by the functional risk score) and covariates separately. The functional risk score is subsequently assessed in the test set using a Cox models that include both the ion mobility specific functional risk score as well as traditional CVD risk factors.

### Ethics statement

All study participants in the Malmö Prevention Project provided written informed consent. The Ethics committee of the Medical Faculty of Lund University approved the study.

## Results

### Example: The Malmö Prevention Project (MPP)

**Fig 1:**
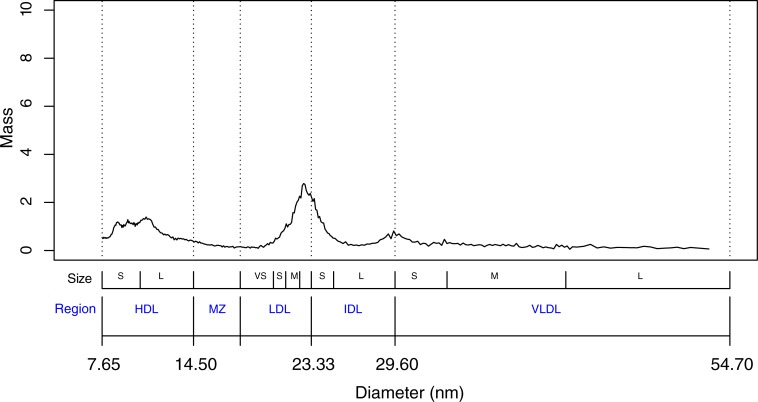
Example profile. Solid line represents particle mass for a representative sample lipoprotein profile. Sizes: (VS = very small; S = small; M = Medium; L = Large). Regions: HDL = High Density Lipoprotein; MZ = Midzone; LDL = Low Density Lipoprotein; IDL = Intermediate Density Lipoprotein; VLDL = Very Low Density Lipoprotein.

The study population was a case-cohort study of 5768 participants derived from the Malmö Prevention Project (MPP), a prospective study of 18,240 participants who were followed for CVD events over a median of 8 years and who had lipoprotein sub-fraction data measured from blood samples drawn at their baseline visit[19, 20]. The data consists of a vector of particle mass measurements for each of 311 particle diameters ranging from 7.65 nm to 54.7 nm as well as CVD event status, time to event, and the following additional baseline covariates: age (years), sex, smoking status, hypertension status, diabetes status, HDL-C, LDL-C, triglycerides, body mass index (BMI), current physical activity (1 = mainly sedentary, 2 = moderate leisure time exercise, 3 = regular leisure time exercise, 4 = hard exercise/competitive sports), and weekly alcohol consumption (total centiliters of pure alcohol assuming alcohol by volume is 5% for beer, 13% for wine and 40% for liqour). The lipoprotein data span size regions that include particles classified as HDL, LDL, IDL, and VLDL respectively, as well as a region denoted as the midzone that comprises particles larger than HDL but smaller than LDL. Each of these regions can be further subdivided into size classes, for example, VLDL-small, -medium and -large. Typical features of a lipoprotein mass profile include a tall, narrow peak in the LDL region (18 to 23 nm) and a short, broad peak in the HDL region (7.65 to 14.5 nm) as shown in a representative sample profile (Fig 1).

The participants were randomly divided into equal sized training and test sets (N = 2884 each) containing 857 and 898 events respectively. Demographic and clinical characteristics of the training and test sets did not differ significantly (Table 1).

**Table 1:** Subject characteristics in train and test set.

	Train	Test	P value*
N	2884	2884	
Age, years (SD)	69.2 (6.0)	69.2 (6.1)	0.88
Male, n (%)"	1949 (67.6)	1993 (69.1)	0.22
CVD Event, n (%)	857 (29.7)	898 (31.1)	0.25
Smoking, n (%)	570 (19.8)	575 (19.9)	0.89
Hypertension, n (%)	2103 (72.9)	2133 (74.0)	0.39
Prevalent Diabetes, n (%)	418 (14.5)	450 (15.6)	0.25
Triglycerides, mmol/L (SD)	1.26 (0.64)	1.25 (0.62)	0.80
HDL-C, mmol/L (SD)	1.38 (0.40)	1.38 (0.40)	0.96
LDL-C, mmol/L (SD)	3.73 (0.96)	3.72 (0.96)	0.62
BMI, kg/m^2^ (SD)	27.1 (4.1)	27.1 (4.1)	0.88
Physical Activity, n (%)			0.39
Mainly sedentary	267 (10.3)	278 (10.6)	
Moderate leisure time exercise	1888 (72.9)	1884 (72.2)	
Regular leisure time exercise	432 (16.7)	442 (16.9)	
Hard exercise/competitive sports	2 (0.1)	7 (0.3)	
Weekly Alcohol, cl (SD)	12.6 (15.9)	12.2 (14.8)	0.36

Values are means when SD is provided

*T-test for numeric variables and Chi-square test for categorical variables

**Fig 2:**
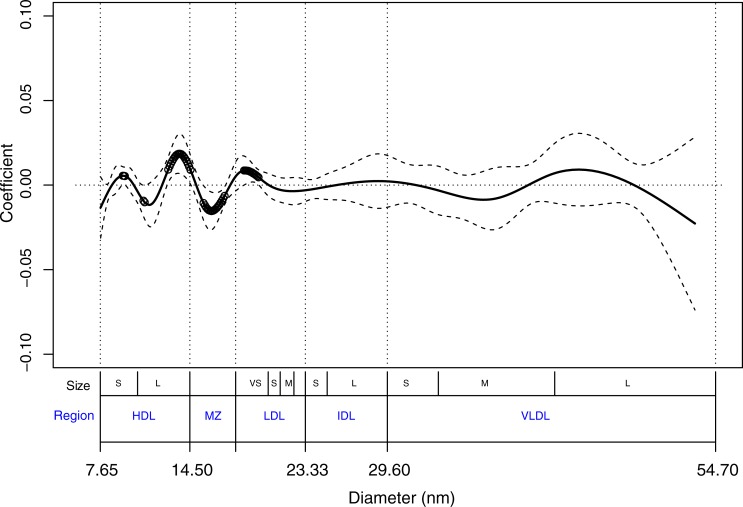
Estimated Cox regression coefficients across ion mobility profile. Solid line represents estimated regression coefficients. Dashed lines represent upper and lower 95% confidence intervals for regression coefficients. Points indicate regions of significance (95% CI does not include zero). Sizes: (VS = very small; S = small; M = Medium; L = Large) Regions: HDL = High Density Lipoprotein; MZ = Midzone; LDL = Low Density Lipoprotein; IDL = Intermediate Density Lipoprotein; VLDL = Very Low Density Lipoprotein.

An evaluation of the power to detect a variety of potential true coefficient functions through simulations (S2 Fig) showed that high power and low false positive rates often co-occurred when degrees of freedom ranged between 7 and 16. We therefore chose the nested spline series with 8 and 15 degrees of freedom for determining smoothness. Natural cubic splines with 8 and 15 degrees of freedom as a function of particle diameter were applied to the matrix of lipoprotein particle mass measurements from the training set (2884 subjects each having 311 particle measurements), resulting in matrices of reduced dimension (2884 subjects by 8 or 15 basis vectors) to represent the particle measurements for modeling purposes. Cox models to predict time to CVD event as a function of the dimension reduced particle matrix were fit to the training set, along with other covariates including age, sex, smoking, hypertension, prevalent diabetes, and baseline levels of LDL-C, HDL-C, and triglycerides. The model with 15 degrees of freedom was selected based on a likelihood ratio test (p = 0.009) comparing the models with 8 and 15 degrees of freedom. A plot (Fig 2) of the estimated coefficient function, $\hat{\beta }(s)$, and corresponding 95% confidence interval versus particle diameter showed that particles with the following diameter ranges were associated with increased risk: 9.375 to 9.525 nm (HDL small), 12.85 to 14.55 nm (HDL Large) and 18.65 to 19.75 nm (LDL very small); particles with diameters ranging from 10.95 to 11.05 nm (HDL large) and 15.55 to 17.15 nm (midzone) were associated with decreased risk.

A natural cubic spline with 15 degrees of freedom as a function of particle diameter was then applied to the matrix of lipoprotein particle mass measurements from the test set (2884 subjects each having 311 particle measurements), resulting in a matrix of reduced dimension (2884 subjects by 15 basis vectors) to represent the particle measurements for test set subjects. A functional risk score for each test set subject was then calculated based on a linear combination of the 15 particle mass specific regression coefficients estimated from the training set Cox model and the row of the dimension reduced particle matrix corresponding to the test set subject. The time to CVD event in the test set subjects was analyzed in a Cox model (Model 1) that included the functional risk score as well as age, sex, smoking, hypertension, prevalent diabetes, and baseline levels of LDL-C, HDL-C, and triglycerides. The functional risk score was associated with increased CVD risk (HR per SD = 1.22 (95% CI: 1.12 to 1.33); p < 0.0001). In a similar model where time to CVD was modeled as a function of quartiles of the functional risk score, being in a higher risk quartile was also associated with a higher risk of a CVD event. Since measurements of BMI, physical activity, and alcohol consumption were not available for all subjects, a second model (Model 2) that included these variables as well as all Model 1 variables was fit to the test set subjects having no missing data (N = 2611). The functional risk score remained associated with increased CVD risk after additional adjustment for BMI, physical activity, and alcohol consumption (HR per SD = 1.21 (95% CI: 1.10 to 1.32); p < 0.0001) (Table 2).

**Table 2:** Cox model results of time to CVD event among test set subjects.

		Model 1				Model 2			
Model	Variable	Hazard Ratio	95% CI		P value	Hazard Ratio	95% CI		P value
Continuous	Functional Risk Score (SD)	1.22	1.12	1.33	< 0.0001	1.21	1.10	1.32	< 0.0001
	Age (years)	1.04	1.03	1.05	< 0.0001	1.04	1.02	1.05	< 0.0001
	Male	1.43	1.22	1.68	< 0.0001	1.47	1.23	1.77	< 0.0001
	Smoker	1.33	1.13	1.56	0.0006	1.21	1.01	1.45	0.04
	HTN	1.30	1.1	1.53	0.002	1.32	1.11	1.58	0.002
	Diabetes	1.48	1.25	1.76	< 0.0001	1.44	1.19	1.74	0.0002
	HDL-C	0.88	0.72	1.08	0.22	0.93	0.74	1.17	0.54
	LDL-C	1.26	1.17	1.37	< 0.0001	1.26	1.15	1.37	< 0.0001
	Triglycerides	0.91	0.8	1.03	0.14	0.92	0.80	1.05	0.21
	BMI					1.00	0.98	1.02	0.74
	Physical Activity					0.87	0.76	1.00	0.05
	Alcohol					1.00	0.99	1.00	0.27
Quartiles	Q2 vs. Q1	1.25	1.03	1.53	0.026	1.23	1.00	1.52	0.05
	Q3 vs. Q1	1.25	1.02	1.54	0.033	1.24	1.00	1.54	0.05
	Q4 vs. Q1	1.48	1.18	1.86	0.0006	1.34	1.05	1.70	0.02
	Age (years)	1.04	1.03	1.05	< 0.0001	1.04	1.02	1.05	< 0.0001
	Male	1.44	1.23	1.69	< 0.0001	1.49	1.24	1.79	< 0.0001
	Smoker	1.32	1.12	1.55	0.0009	1.20	1.00	1.43	0.05
	HTN	1.30	1.1	1.54	0.002	1.33	1.11	1.59	0.002
	Diabetes	1.50	1.26	1.78	< 0.0001	1.46	1.21	1.76	< 0.0001
	HDL-C	0.86	0.7	1.06	0.15	0.91	0.72	1.14	0.40
	LDL-C	1.23	1.13	1.33	< 0.0001	1.21	1.11	1.31	< 0.0001
	Triglycerides	0.95	0.84	1.07	0.41	0.97	0.85	1.11	0.66
	BMI					1.00	0.98	1.02	0.73
	Physical Activity					0.86	0.75	0.99	0.04
	Alcohol					1.00	0.99	1.00	0.31

**Fig 3:**
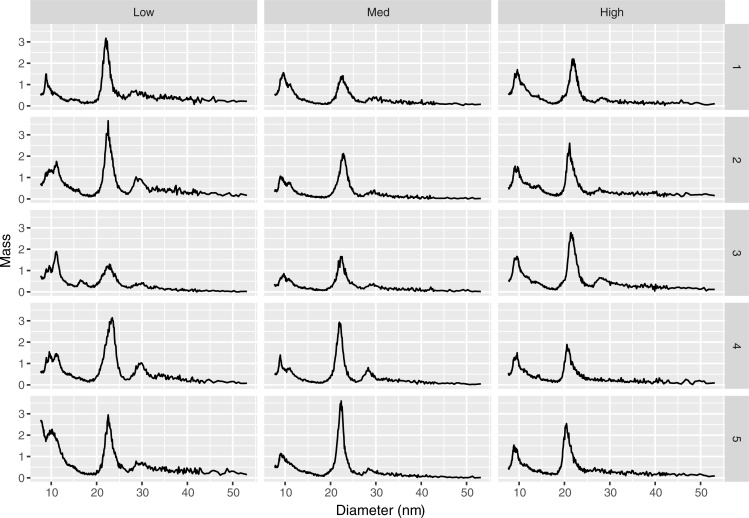
Randomly selected profiles of test set subjects having low (< 2^nd^ percentile), median (49^th^ to 51^st^ percentiles) and high (> 98^th^ percentile) functional risk scores.

To visualize how subject lipoprotein profiles might vary over differing levels of the functional risk score, profiles from five test set subjects having low(< 2^nd^ percentile), median (49^th^ to 51^st^ percentile), or high (> 98^th^ percentile) functional risk scores were randomly selected and plotted (Fig 3). Profile peaks in the LDL region of the low-risk group appear to be both taller and centered at a larger diameter (~23 nm vs. ~21 nm) than those in the high-risk group. In addition, a peak in the IDL region was more apparent in the low-risk profiles than among high-risk profiles. Differences among the peaks in the HDL region across risk groups were also apparent but tended to be more variable across samples.

### Simulations

Simulations were performed under a null model to evaluate type I error and under a variety of alternative hypotheses to evaluate power for four different sample sizes: *N* = 500, 1000, 2500, and 5000. For each *N*, results are summarized for *K =* 500 iterations.

*N* random profiles of particle mass were generated from a multivariate normal distribution using the observed vector of means and variance covariance matrix among the MPP training set as parameters. The random profiles demonstrated features similar to the MPP profiles (S3 Fig).

The log hazard of an event occurring for each generated profile *i*, (*i* = 1,⋯,*N*), was calculated as:

$$\log\;\lambda_{it}=\alpha_{t}+\sum_{j=1}^{J}\beta_{j}(x_{ij}-x_{.j})$$

where *α*_*t*_ is a baseline hazard, *β*_*j*_ is the value of an assumed true coefficient function, *β*(*s*_*j*_), at size *s*_*j*_, and (***x***_*ij*_−***x***_.*j*_) is the particle mass for profile *i* centered around the mean of the *N* generated profile values at size *s*_*j*_ $\left(x_{.j}=\sum_{i=1}^{N}\frac{x_{ij}}{N}\right)$.

Event times were randomly generated assuming an exponential distribution based on the individual specific log-hazards with $\alpha_{t}=-\log\left(\frac{.9}{10}\right)$ such that the expected cumulative incidence of events within a 10-year time period among “mean profiles” (*i*.*e*.(***x***_*ij*_−***x***_.*j*_) = 0 for all *j* = 1,⋯,*J*) = 10%. Event times greater than 10 years were censored.

Given a set of *N* randomly generated profiles and event times, analysis proceeded as described in the methods: Cox models for two choices of degrees of freedom were fit and compared by likelihood ratio test and the selected model was then evaluated by likelihood ratio test versus a null model with Bonferroni adjustment for assessment of two models. If the null model was rejected, 95% confidence intervals for $\hat{\beta }(s)$ were determined and compared with the assumed function, *β*(*s*).

Type I error was assessed by two metrics: the proportion of iterations in which the Bonferroni adjusted p-value from a likelihood ratio test comparing the selected model to a null model was significant (< 0.05), and the proportion of size bins, *s*_*j*_, for which the 95% confidence interval for $\hat{\beta }(s_{j})$ did not contain zero. Power was defined as the proportion of iterations in which the Bonferroni adjusted p-value from the likelihood ratio test rejected the null model (< 0.05), and among those size bins, *s*_*j*_, where the assumed *β*(*s*_*j*_) > 0, we required at least 50% of size bins to have both $\hat{\beta }(s_{j})$ in the same direction as *β*(*s*_*j*_) and a 95% confidence interval for $\hat{\beta }(s_{j})$ that excluded zero.

**Fig 4:**
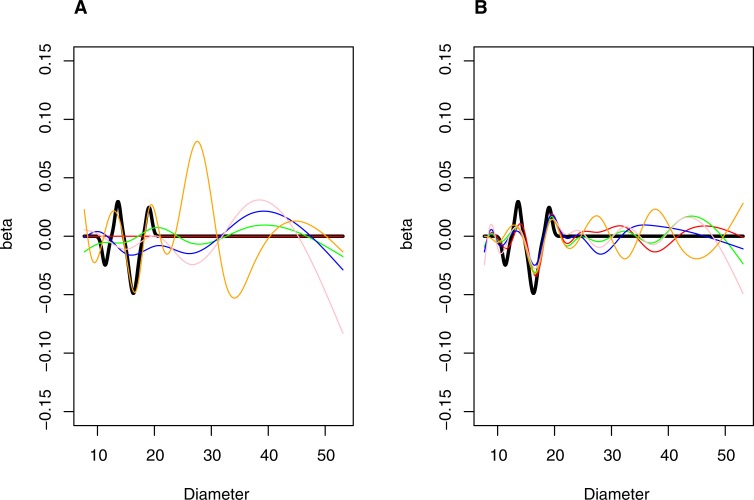
Simulated coefficient profiles under an alternative hypothesis for nested spline series with 7 and 13 degrees of freedom. *N* = 500 (Panel A) and *N* = 5000 (Panel B). Thick (black) line is true profile. Thin (colored) lines are representative simulated profiles.

**Fig 5:**
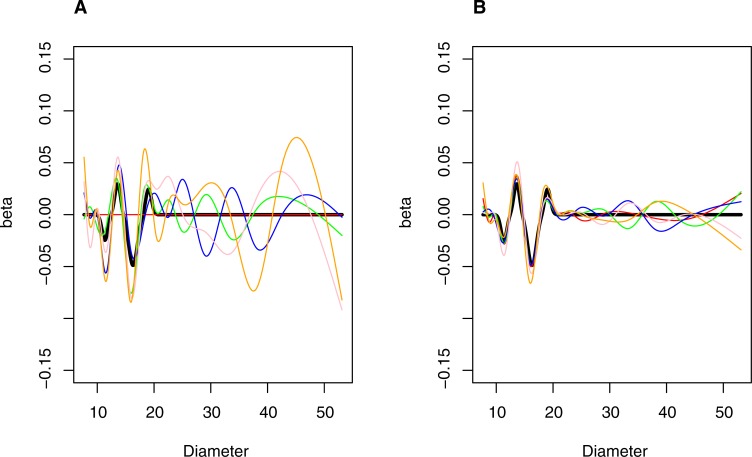
Simulated coefficient profiles under an alternative hypothesis for nested spline series with 8 and 15 degrees of freedom. *N* = 500 (Panel A) and *N* = 5000 (Panel B). Thick (black) line is true profile. Thin (colored) lines are representative simulated profiles.

A function with true effects chosen to have features somewhat similar to those observed in the applied example was chosen as a representative example for summarizing the operating characteristics of the method. Specifically, a function having four regions with effects on risk was generated. Two regions, centered at 13.6 and 19.05 nm, increased risk by log(1.03) and log(1.025) per SD of particle mass at their peak and an additional two regions, centered at 11.35 and 16.3 nm, decreased risk by log(1.025) and log(1.05) per SD of particle mass respectively. The four regions with true effects were generated assuming the log hazard follows a Gaussian distribution centered at the peak and with standard deviations of 0.5, 0.5, 0.75 and 0.5 respectively. Effects of specific distributions were forced to zero at +,- 3 SD from the mean. In cases where more than one defined distribution overlapped a particular size bin, the total log hazard of the size bin was calculated as the sum of the effects of the overlapping distributions. The profile of the true effect size for simulations can be visualized by the thick black line in Figs 4 and 5.

Type I error was close to the expected 5% (range: 2% to 5%) for the 7 and 13 as well as the 8 and 15 series. Power was poor (< 25%) for simulations where the average number of events was ≤100. However, it increased with increasing sample size in simulations averaging 500 events: to 83% for the 7 and 13 series and 99% for the 8 and15 series. The median proportion of false positive bins was in a generally acceptable range (7% to 15% for the 7 and 13 series and 2% to 5% for the 8 and 15 series). As sample size increased, the estimated function approached the true function, particularly for the 8 and 15 series, as demonstrated visually (Figs 3 and 4), and by the increasing proportion of true-positive size bins. In simulations averaging 50 events, the median proportion of true positive bins were 25% for the 7 and 13 series and 23% for the 8 and 15 series; in simulations averaging 500 events, these proportions increased to 67% for the 7 and 13 series and 73% for the 8 and 15 series (Table 3).

**Table 3:** Simulation results.

			Null Model		Alternative Model		
Nested Series	N	Events	Type I error*	False Positive Rate**	Power***	False Positive Rate**	True Positive Rate****
7, 13	500	50	0.042	0 (0 : 0)	0.030	0.07 (0 : 0.21)	0.25 (0.04 : 0.31)
	1000	99	0.028	0 (0 : 0)	0.116	0.15 (0.06 : 0.31)	0.35 (0.29 : 0.42)
	2500	249	0.024	0 (0 : 0)	0.406	0.12 (0.06 : 0.23)	0.46 (0.4 : 0.60)
	5000	502	0.028	0 (0 : 0)	0.836	0.15 (0.1 : 0.21)	0.67 (0.57 : 0.70)
8, 15	500	50	0.054	0 (0 : 0)	0.100	0.02 (0 : 0.13)	0.23 (0.05 : 0.36)
	1000	100	0.042	0 (0 : 0)	0.248	0.05 (0 : 0.12)	0.37 (0.28 : 0.50)
	2500	249	0.034	0 (0 : 0)	0.794	0.03 (0 : 0.10)	0.62 (0.53 : 0.68)
	5000	500	0.040	0 (0 : 0)	0.996	0.02 (0 : 0.09)	0.73 (0.69 : 0.75)

* Based on likelihood ratio test for selected degrees of freedom (alpha = 0.05)

** Median (IQR) of the proportion of true negative bins with 95% CI of beta coefficients excluding zero

*** Based on a likelihood ratio test p-value < 0.05 AND at least 50% of true positive bins with 95% CI excluding zero

**** Median (IQR) of the proportion of true positive bins with 95% CI of beta coefficients excluding zero

## Discussion

A high resolution size distribution of lipoprotein particles can be measured by ion-mobility and other methods. However, lipoprotein measurements are typically reported as particle concentration in a small number of large regions with broad size range. In this study, we developed and assessed methods that can assess the association of the entire lipoprotein size spectrum as a single score. This approach has the advantage that it does not arbitrarily discard or collapse information generated by ion mobility measurement of lipoprotein size distribution. In principle, this approach can also be applied to other methods that analyze lipoprotein size or density in a continuous manner.

In the applied example, the functional model fit to the training set allowed calculation of a functional risk score for test set subjects. Evaluation of the score in the test set confirmed that the model differentiated subjects according to risk of CVD events. When ranked by quartile of functional risk score, the hazard for CVD among the highest quartile of the risk score was 48% higher than among subjects in the lowest quartile.

In addition, two types of graphs assisted with interpretation of the model. A plot of the estimated coefficient function indicated that after accounting for effects of traditional risk factors such as age, sex, smoking, diabetes, hypertension, LDL-C, HDL-C and triglycerides, higher levels of large HDL particles (12.85 to 14.55 nm) and very small LDL particles (18.65 to 19.75 nm) were associated with increased CVD risk. In contrast, higher levels of particles in a region of unknown classification, termed the midzone (15.55 to 17.15 nm), were associated with decreased CVD risk. Visual inspection of lipoprotein mass profiles among patients selected from the high and low extremes of the functional risk score, indicate that the peak diameter size in the LDL region among the high risk profiles is shifted towards somewhat smaller particle diameters. The association of the small LDL particles and large HDL particles with higher risk of CVD is consistent with trends found in previous studies[13, 21]. The effects found for the particles of unknown classification (midzone) appear to be novel and should be followed up in additional cohorts.

The simulated results demonstrate that type I error was well controlled for the 7 and 13 as well as the 8 and 15 series of splines when evaluating a null model. Furthermore, the functional model converged toward the underlying true functional form under both a null model as well as a fairly complex model having four regions with true effects. Under the alternative hypothesis, the proportion of bins with true effects that the models correctly identified increased with sample size and power was greater than 80% for both spline series in simulations averaging 500 events. Thus, the MPP training set (857 events) is of sufficient size to detect similar underlying true effects with good power.

There are several advantages of the functional analysis approach when compared to a previous publication of the same MPP study population that assessed the association of CVD with each of 15 pre-defined diameter subclasses separately [18]. That study reported that two adjacent subclasses of very small LDL particles (19.9 to 20.49 and 20.49 to 20.82 nm) were associated with increased risk of CVD events: hazard ratios for subjects in the highest vs lowest quartiles of these subclasses were 1.33 and 1.26, respectively. The first advantage is that estimation of the coefficient function corresponding to each diameter precludes the need to define size regions. Or, in this example where regions had already been defined, estimation of the coefficient function allows assessment of whether the magnitude and direction of the estimated effects are consistent throughout a defined region. For example, the functional model suggests that effects within the HDL-large region change from risk-decreasing to risk-increasing as diameter increases, whereas these opposite effects likely cancel each other out when a single risk estimate for the entire region is estimated. Second, the functional approach accounts for correlations among the different size regions and provides each subject with a single functional risk score that incorporates risk from the entire range of particle diameters. For example, in the previous analysis, the risk estimates for the two significant very small LDL regions are adjusted for traditional risk factors but are not adjusted for each other or for other lipoprotein regions leaving the reader unsure how to estimate risk for a particular subject. Finally, by plotting the lipoprotein profiles of subjects with very low and very high functional risk scores, it was possible to discern distinguishing features of such profiles.

Disadvantages of the functional analysis include the need to choose the smoothness parameter for the functional model (degrees of freedom). We initially explored a bootstrap approach but found it had inflated type I error. Instead, we proposed the nested spline method which allowed proper control of type I error in exchange for somewhat reduced power. Power will depend on the underlying true function, the particular series of nested splines chosen, and the number of choices for degrees of freedom allowed in the chosen series. To select series that appeared to have robust operating characteristics, we first simulated a variety of potential true functions and evaluated results over a wide range of degrees of freedom. For data analysis, we limited to two the number of choices for degrees of freedom in the selected series in order to reduce the penalty of multiple testing as well as to simplify the choice of the best model in the series.

A limitation of the model in the MPP cohort is that we assume relative changes in the hazard of CVD among males and females are of equal magnitude. Since more than 70% of events in the MPP sample occurred in males, the functional model is heavily weighted towards males and may not be equally effective among females, particularly given known gender differences with respect to CVD risk.

Future work in modeling the effect of lipoprotein profiles on risk of CVD includes evaluation of gender specific effects as well as attempting to replicate our proposed functional model from the MPP cohort in additional cohorts. Future methodological work includes investigating additional methods for choosing the degrees of freedom and estimating confidence limits for the functional model that control the false positive rate while maintaining good power.

In conclusion, we have demonstrated the potential utility of a functional model for data generated by methods such as ion mobility. Clinical practice could potentially be improved in several ways by the study results. First, the analysis provided a graphical representation of the coefficient function that may result in improved understanding of the underlying diameter regions most associated with risk of CVD. Second, the functional model provides a single measure of risk due to lipoprotein particle abundance (and possibly other covariates) that could simplify reporting of risk. Finally, in the MPP test set, high functional risk scores were associated with increased risk of CVD after adjustment for established risk factors, indicating that this approach, if validated in additional cohorts, has the potential to improve CVD risk assessment.

## Supporting information

S1 Fig Location of knots for nested splines having 8 and15 degrees of freedom. (PDF)

S2 Fig Simulated results by degree of freedom for ten different true functions. A (upper left): Regression coefficients of true function; B (upper right): Power; C (lower left): Proportion of false positive bins; D (lower right): Proportion of True positive bins. Red, black, blue and green lines show results from simulations with sample size of 500, 1000, 2500 and 5000 respectively.
(PDF)

S3 Fig Observed and simulated profiles. A) Ten randomly chosen profiles from MPP training set. B) Ten simulated profiles generated from multivariate normal distribution.
(PDF)
