# Supplementary figures and images for: Association of cardiovascular events and lipoprotein particle size: Development of a risk score based on functional data analysis
Source: PLoS One. 2019 Mar 7;14(3):e0213172. doi: 10.1371/journal.pone.0213172 (PMC6405139; doi:10.1371/journal.pone.0213172)

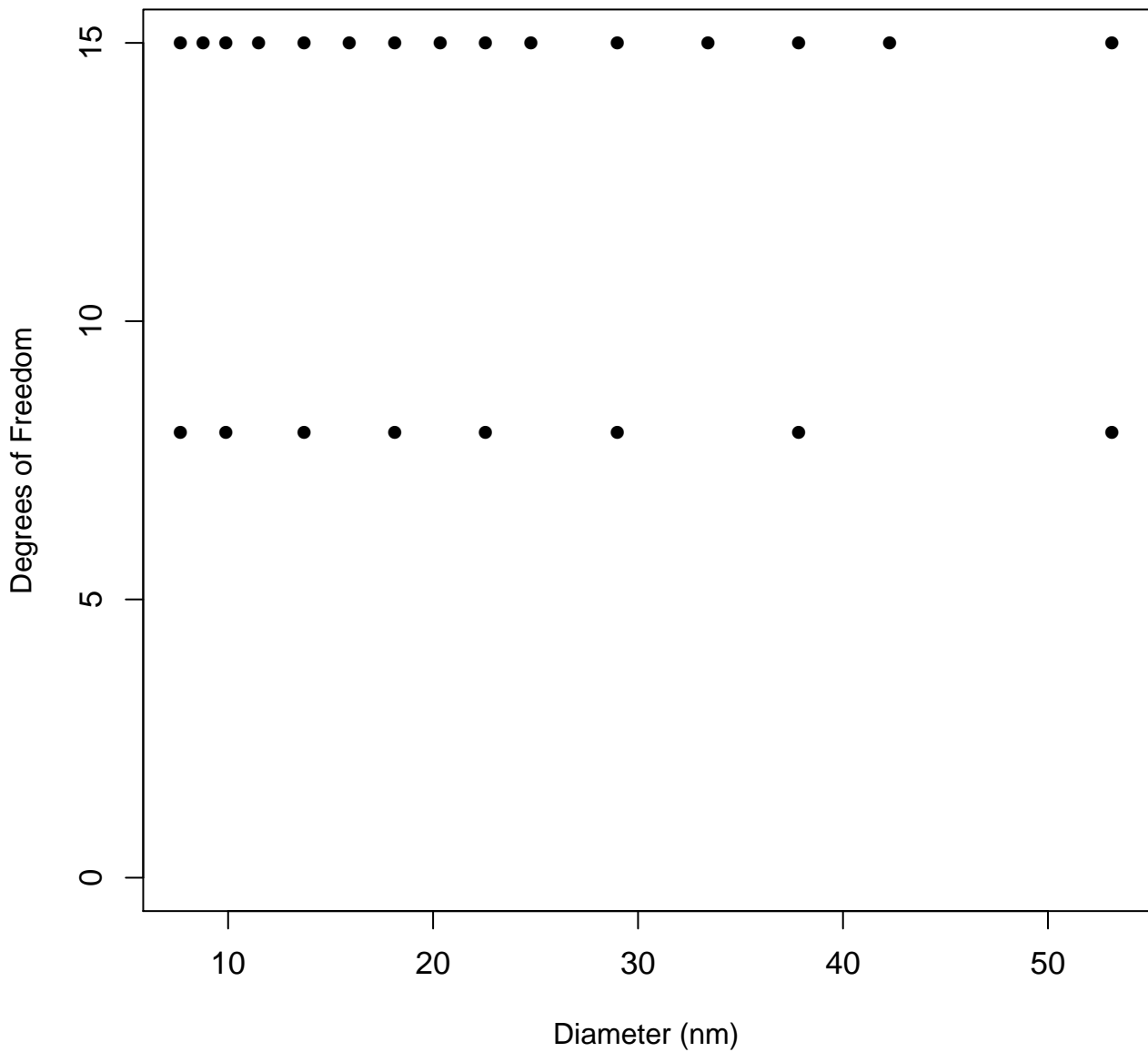

Supplement: S1 Fig — (PDF) [file pone.0213172.s001.pdf]

**A**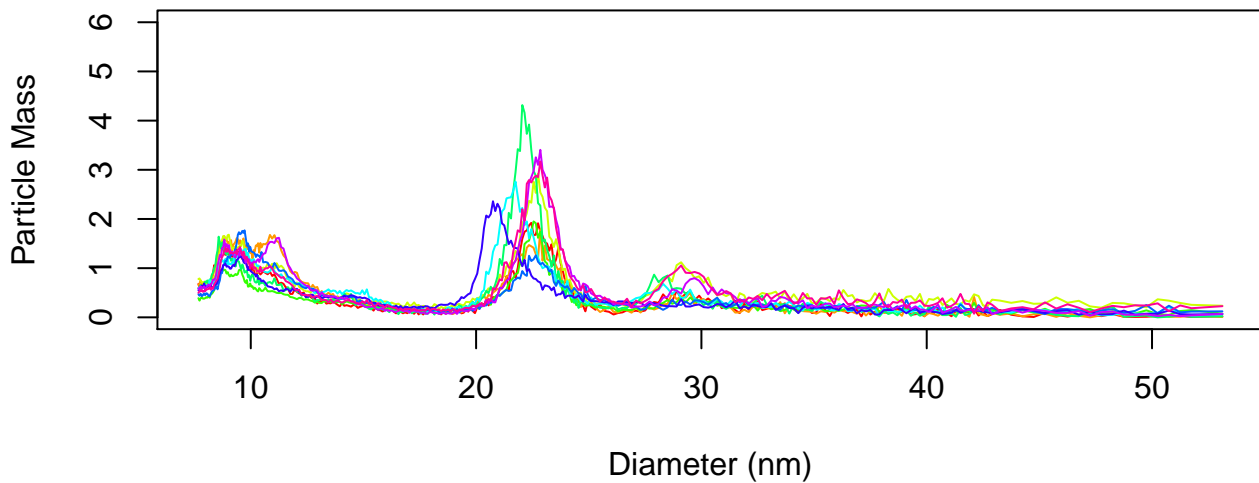**B**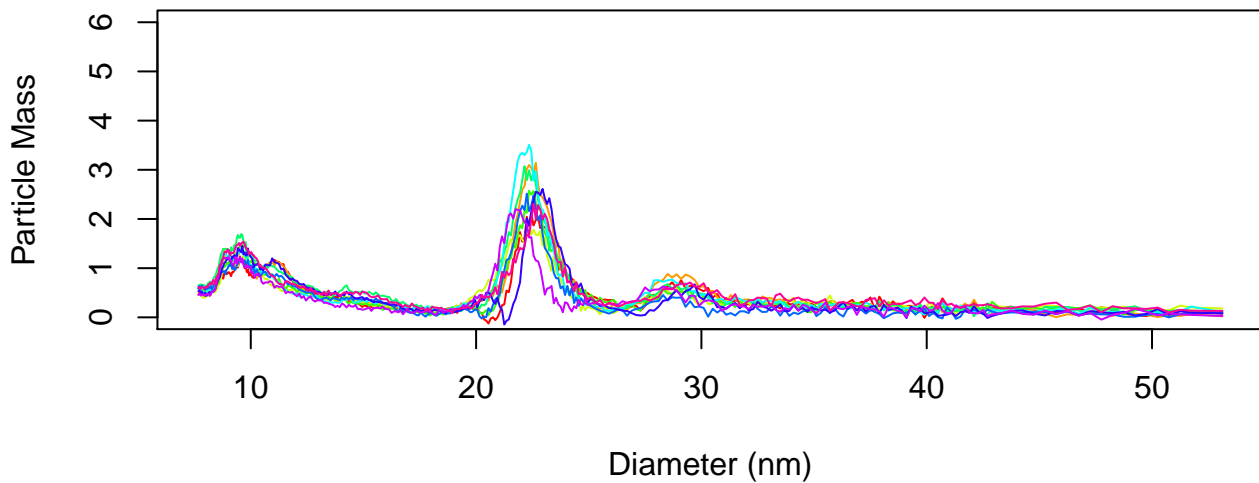

Supplement: S3 Fig — A) Ten randomly chosen profiles from MPP training set. B) Ten simulated profiles generated from multivariate normal distribution. (PDF) [file pone.0213172.s003.pdf]
