# Supplementary material for: Association of cardiovascular events and lipoprotein particle size: Development of a risk score based on functional data analysis
Source: PLoS One. 2019 Mar 7;14(3):e0213172. doi: 10.1371/journal.pone.0213172 (PMC6405139; doi:10.1371/journal.pone.0213172)
Supplement: S2 Fig — A (upper left): Regression coefficients of true function; B (upper right): Power; C (lower left): Proportion of false positive bins; D (lower right): Proportion of True positive bins. Red, black, blue and green lines show results from simulations with sample size of 500, 1000, 2500 and 5000 respectively. (PDF) [file pone.0213172.s002.pdf]

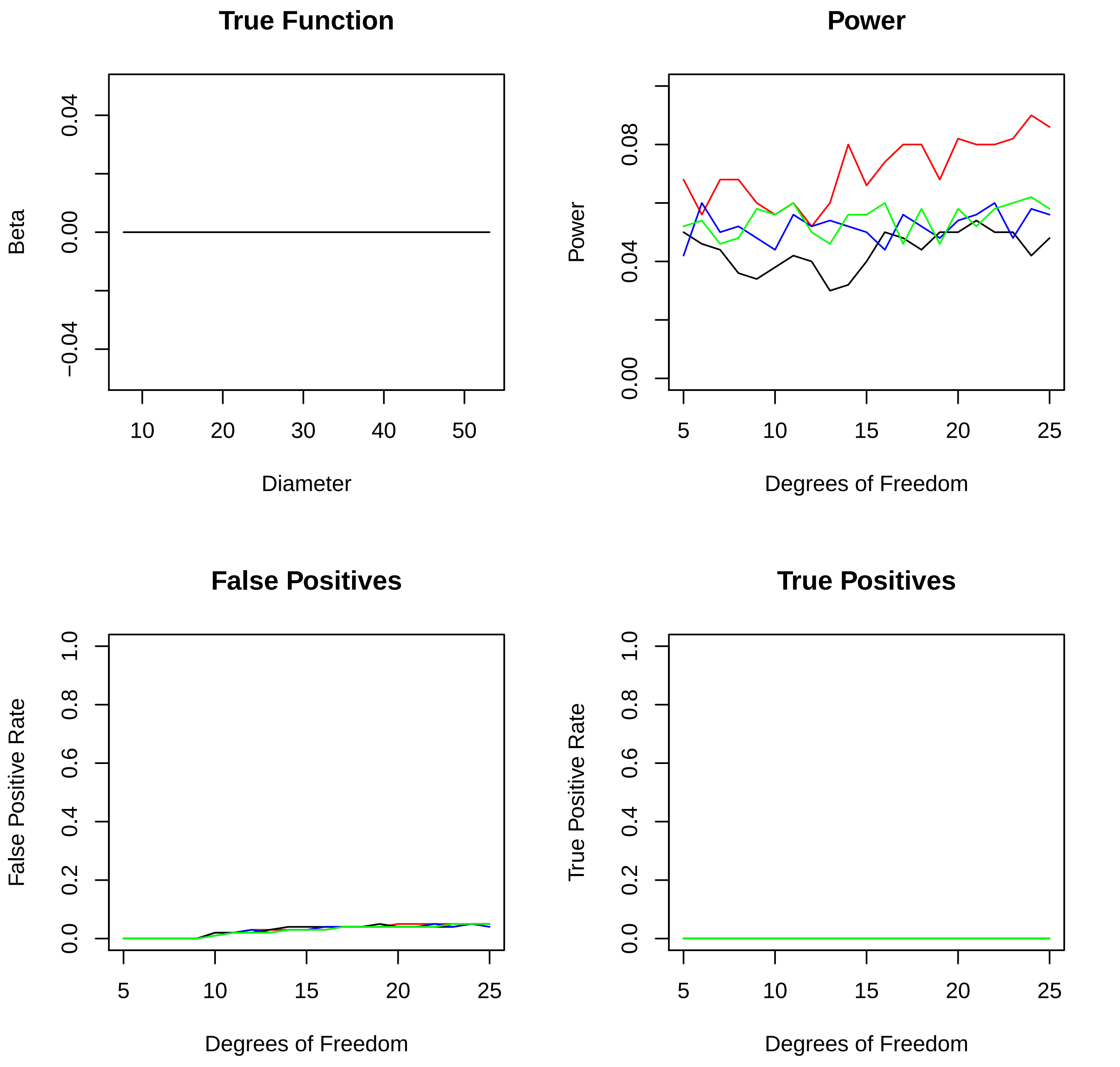

True Function
Beta
Diameter
Power
Power
Degrees of Freedom
False Positives
False Positive Rate
Degrees of Freedom
True Positives
True Positive Rate
Degrees of Freedom

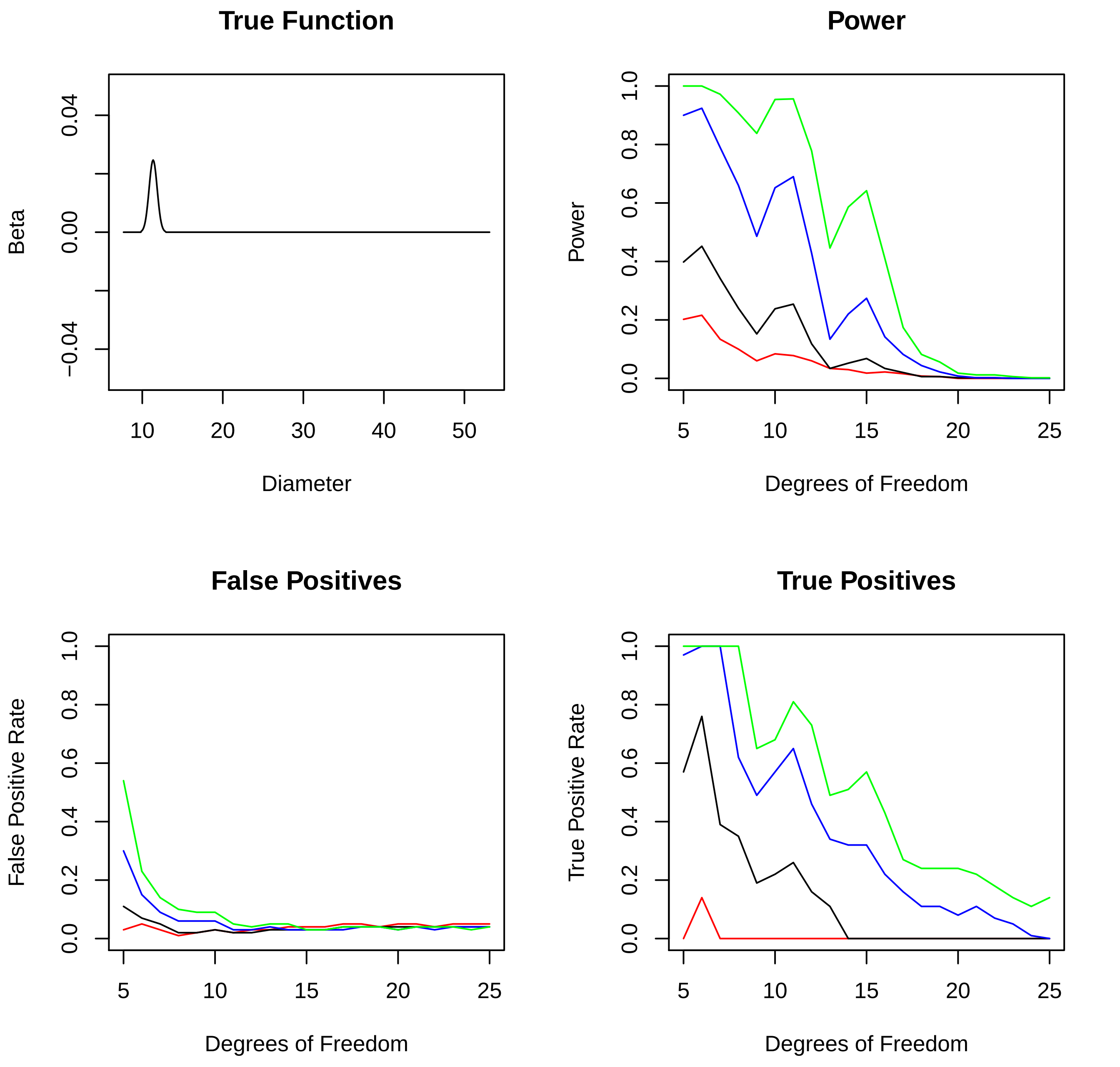
True Function
Beta
Diameter
0.04
0.00
−0.04
10
20
30
40
50
Power
Degrees of Freedom
0.0
0.2
0.4
0.6
0.8
1.0
5
10
15
20
25
False Positives
False Positive Rate
True Positives
True Positive Rate

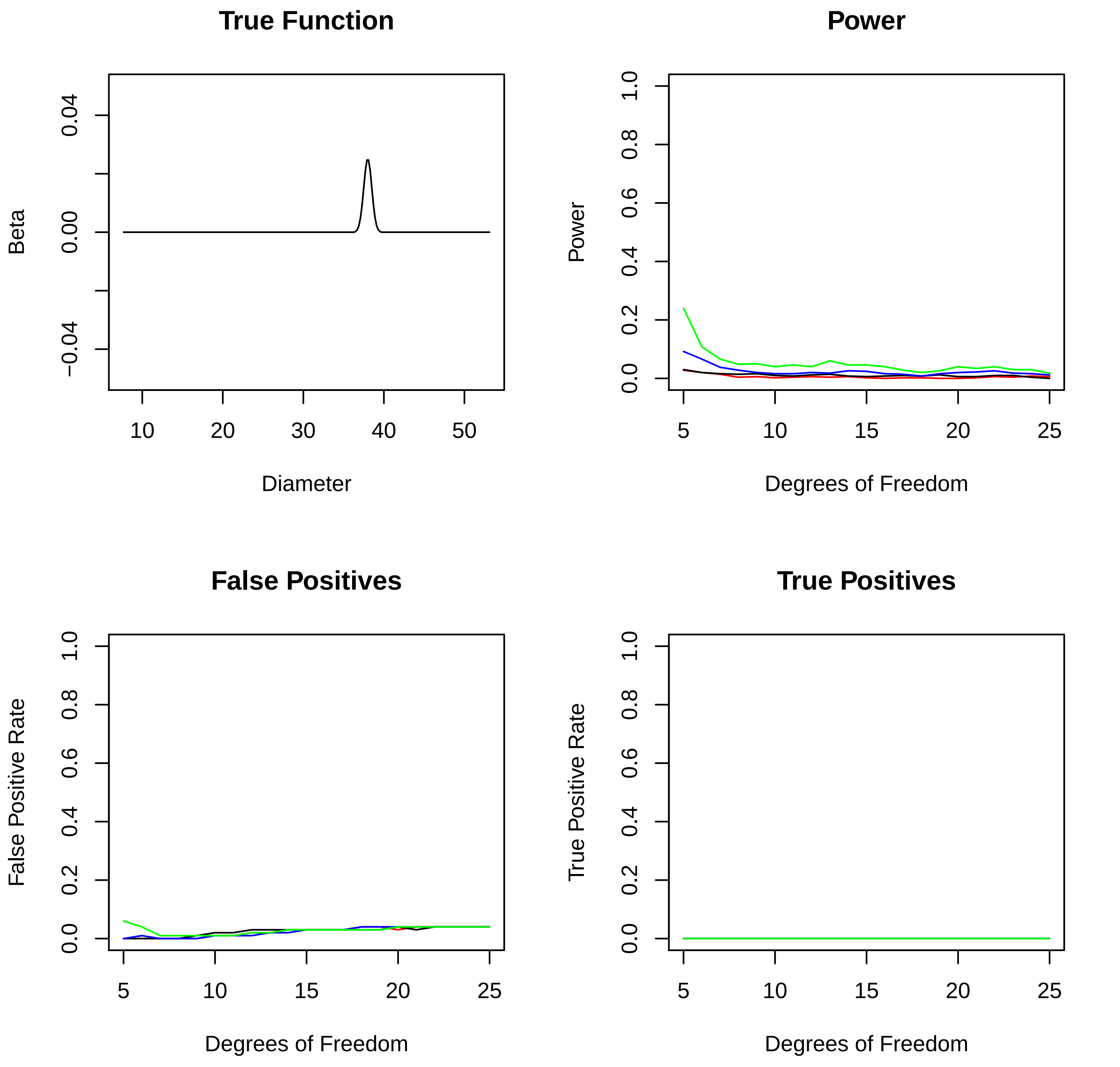
True Function
Beta
0.04
0.00
−0.04
10
20
30
40
50
Diameter
Power
Power
0.0
0.2
0.4
0.6
0.8
1.0
5
10
15
20
25
Degrees of Freedom
False Positives
False Positive Rate
0.0
0.2
0.4
0.6
0.8
1.0
5
10
15
20
25
Degrees of Freedom
True Positives
True Positive Rate
0.0
0.2
0.4
0.6
0.8
1.0
5
10
15
20
25
Degrees of Freedom

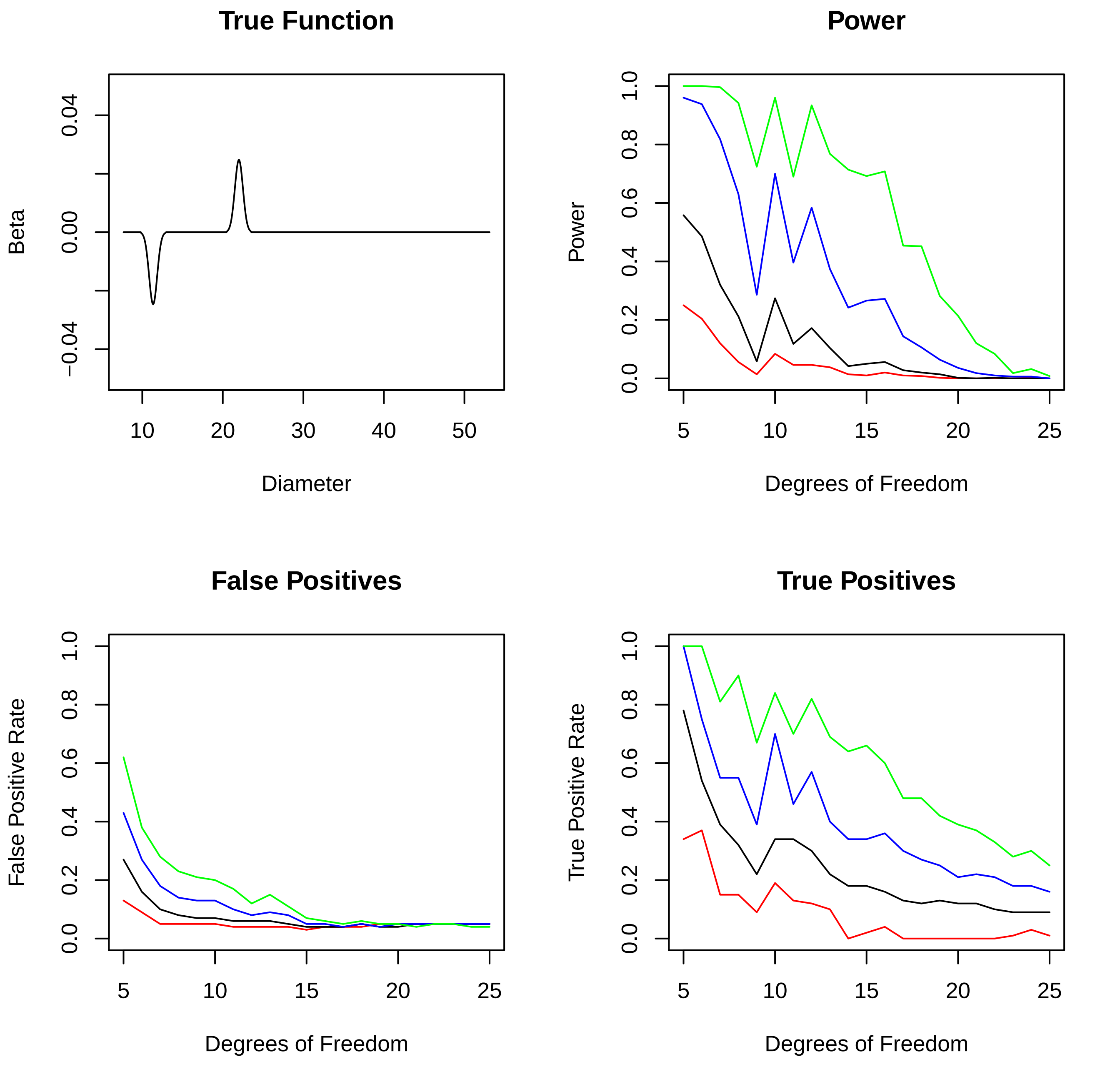
True Function
Beta
Diameter
Power
Degrees of Freedom
False Positives
False Positive Rate
Degrees of Freedom
True Positives
True Positive Rate
Degrees of Freedom

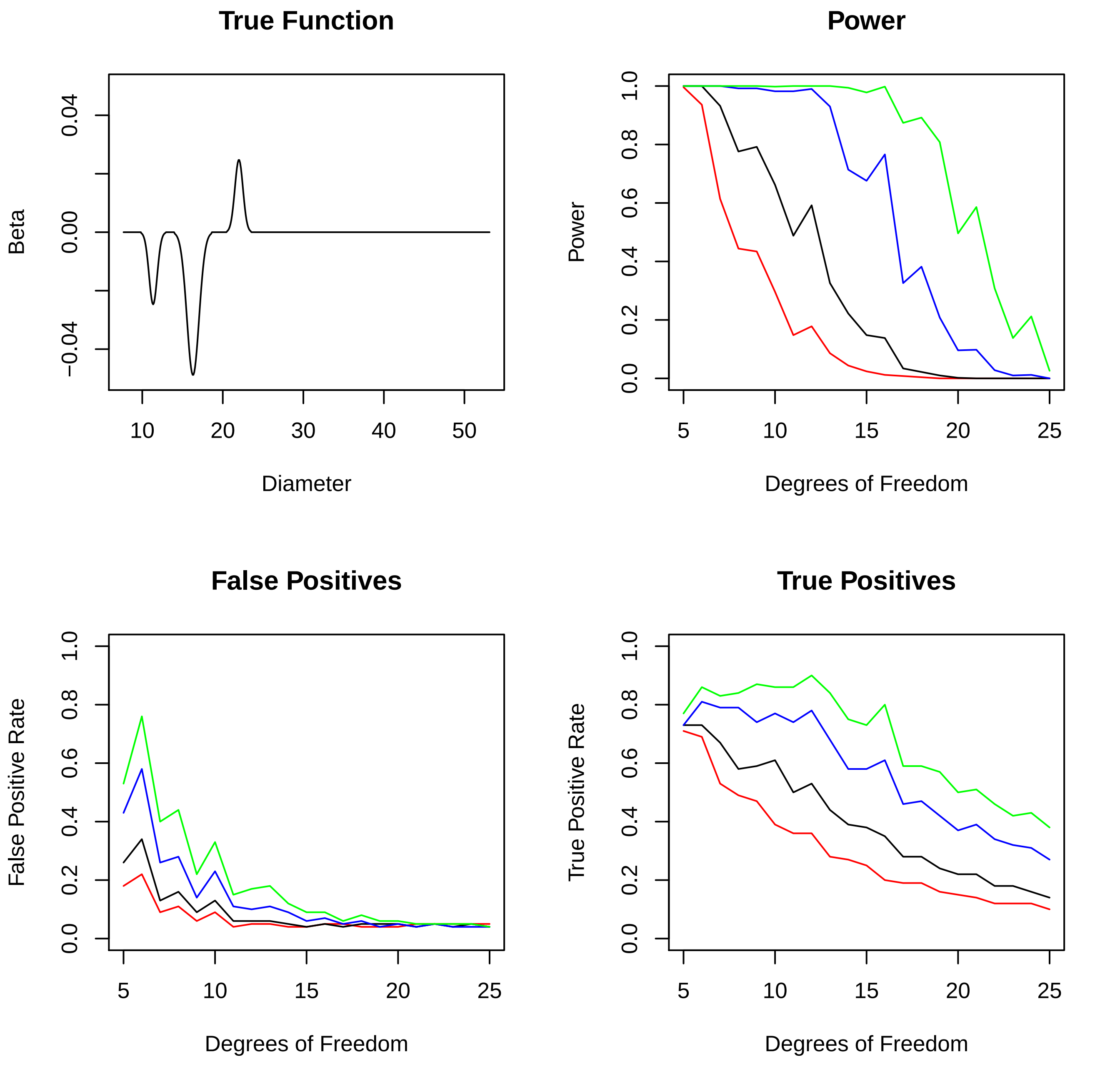
True Function
Beta
Diameter
0.04
0.00
−0.04
10
20
30
40
50
Power
Degrees of Freedom
0.0
0.2
0.4
0.6
0.8
1.0
5
10
15
20
25
False Positives
False Positive Rate
Degrees of Freedom
True Positives
True Positive Rate
Degrees of Freedom

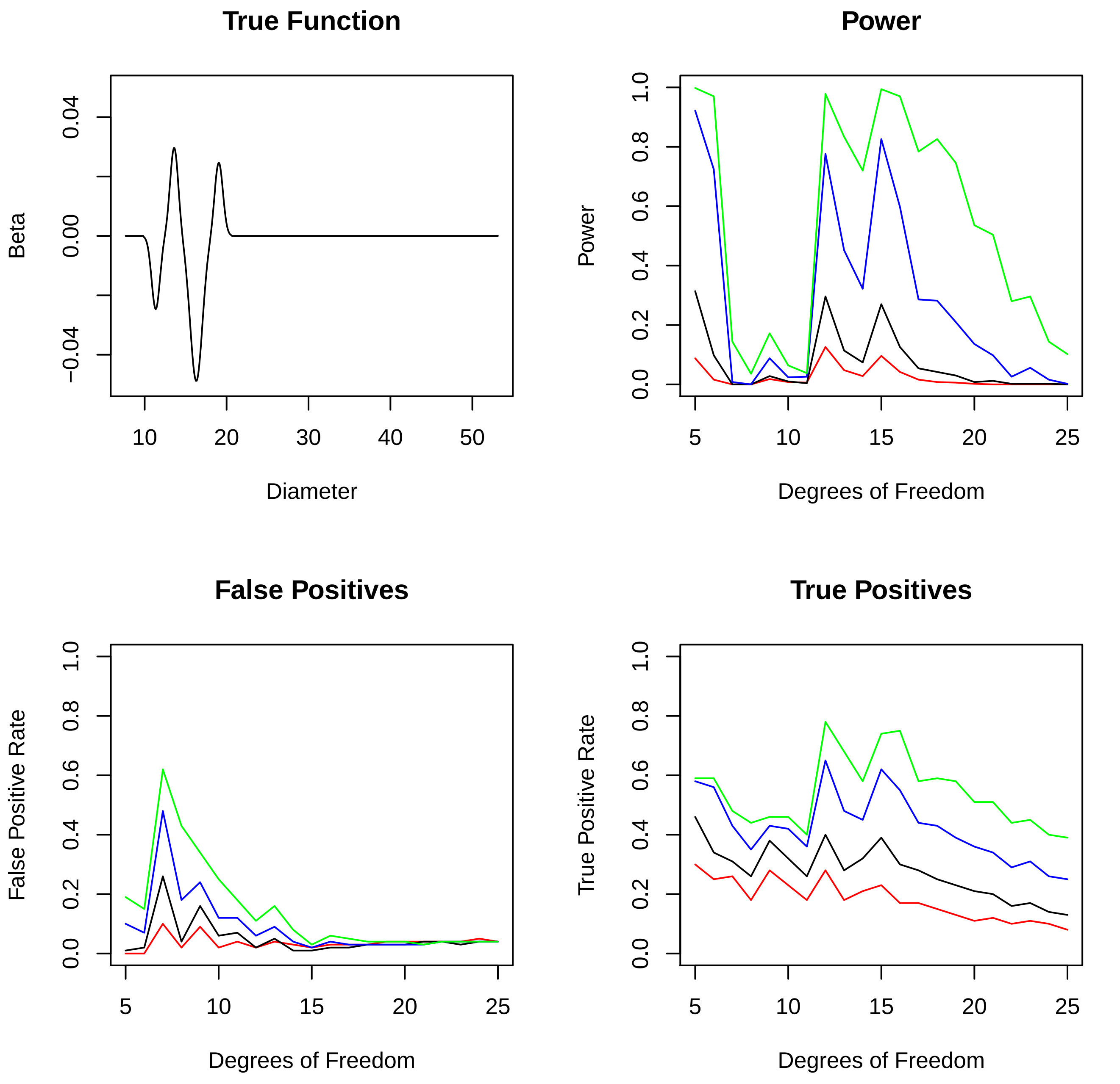
True Function
Power
False Positives
True Positives
Beta
Power
False Positive Rate
True Positive Rate
Diameter
Degrees of Freedom
Degrees of Freedom
Degrees of Freedom

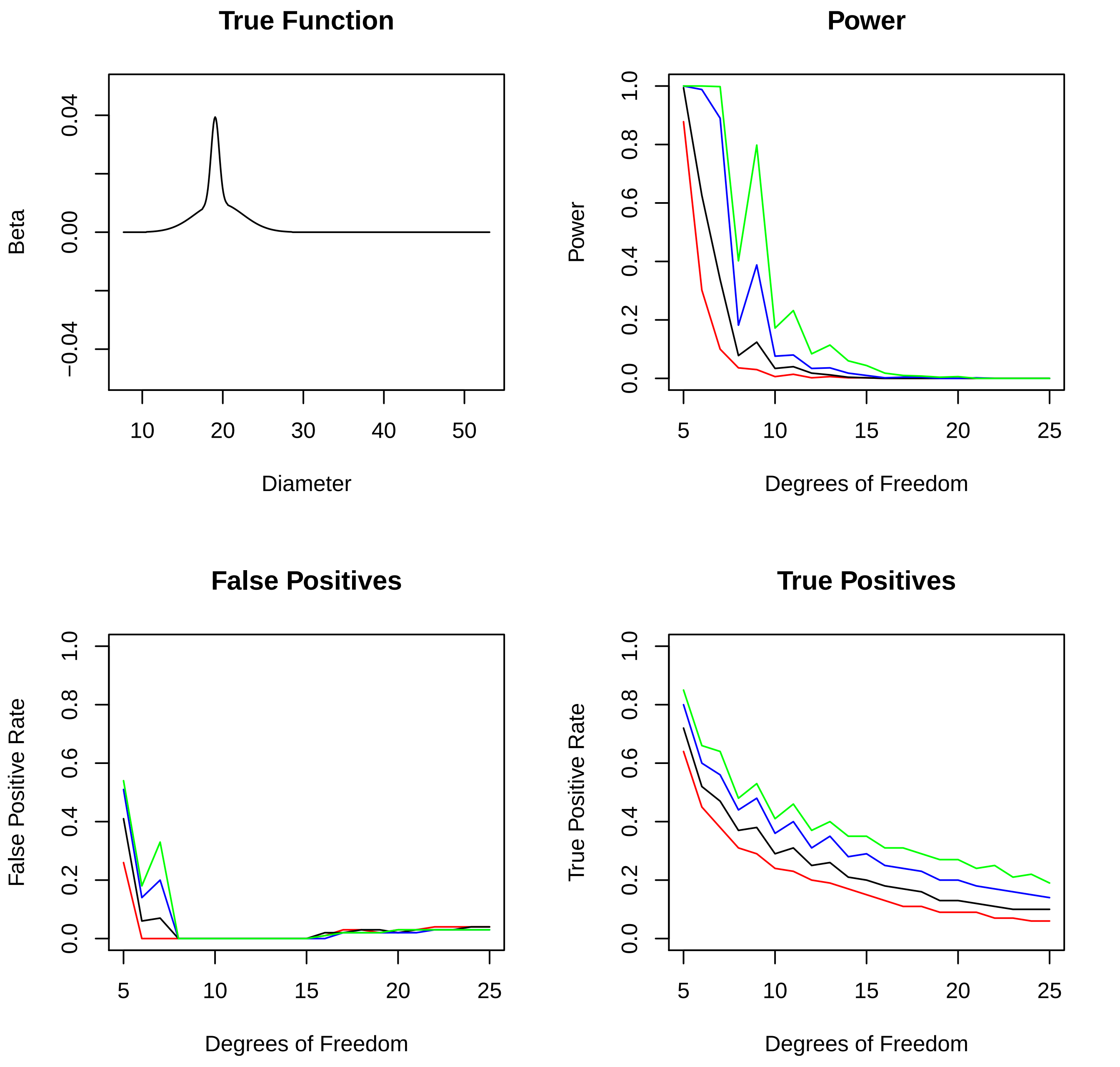
True Function
Beta
0.04
0.00
−0.04
10
20
30
40
50
Diameter
Power
Power
1.0
0.8
0.6
0.4
0.2
0.0
5
10
15
20
25
Degrees of Freedom
False Positives
False Positive Rate
1.0
0.8
0.6
0.4
0.2
0.0
5
10
15
20
25
Degrees of Freedom
True Positives
True Positive Rate
1.0
0.8
0.6
0.4
0.2
0.0
5
10
15
20
25
Degrees of Freedom

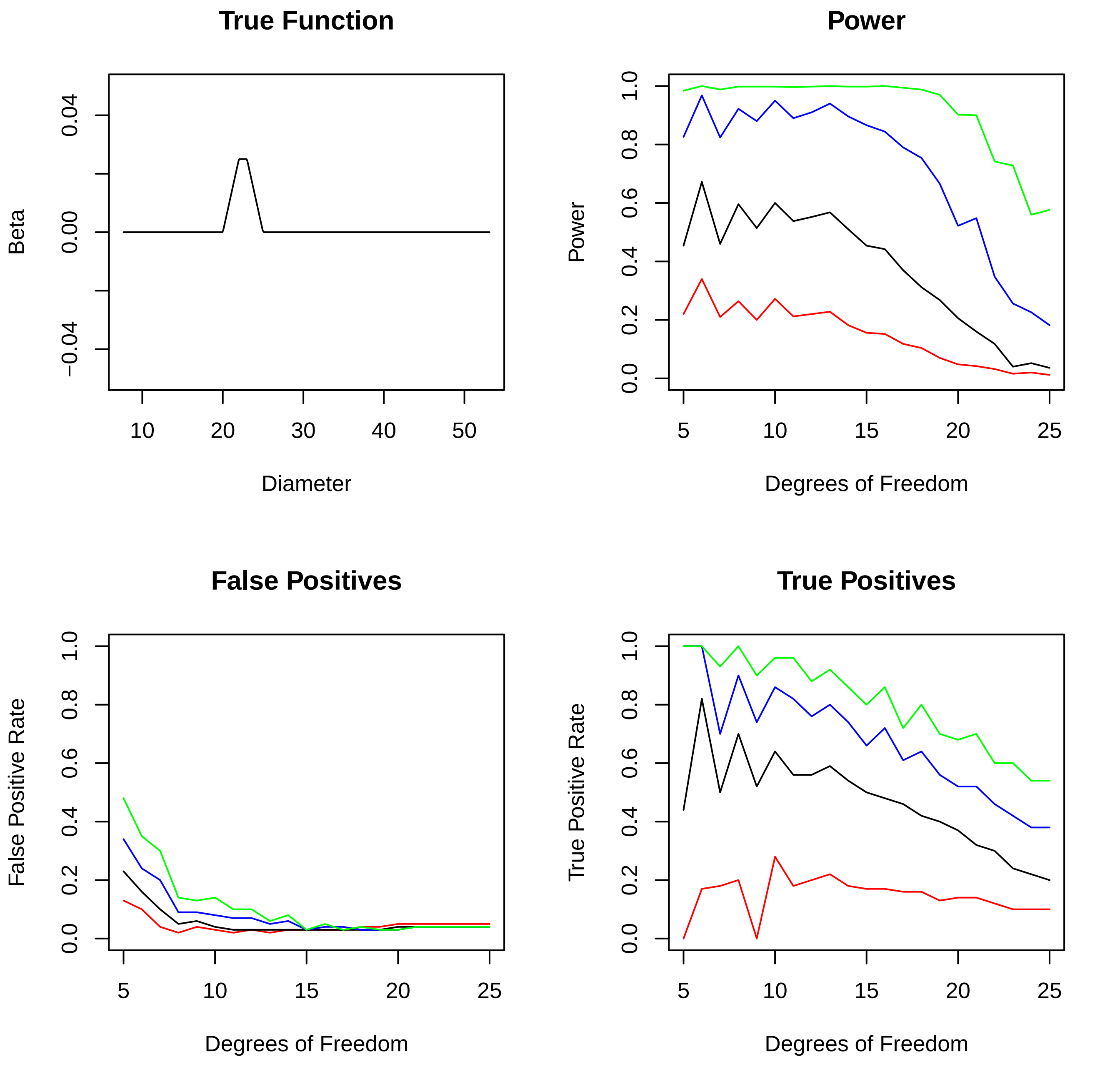
True Function
Beta
0.04
0.00
−0.04
10
20
30
40
50
Diameter
Power
Power
0.0
0.2
0.4
0.6
0.8
1.0
5
10
15
20
25
Degrees of Freedom
False Positives
False Positive Rate
Degrees of Freedom
True Positives
True Positive Rate
Degrees of Freedom

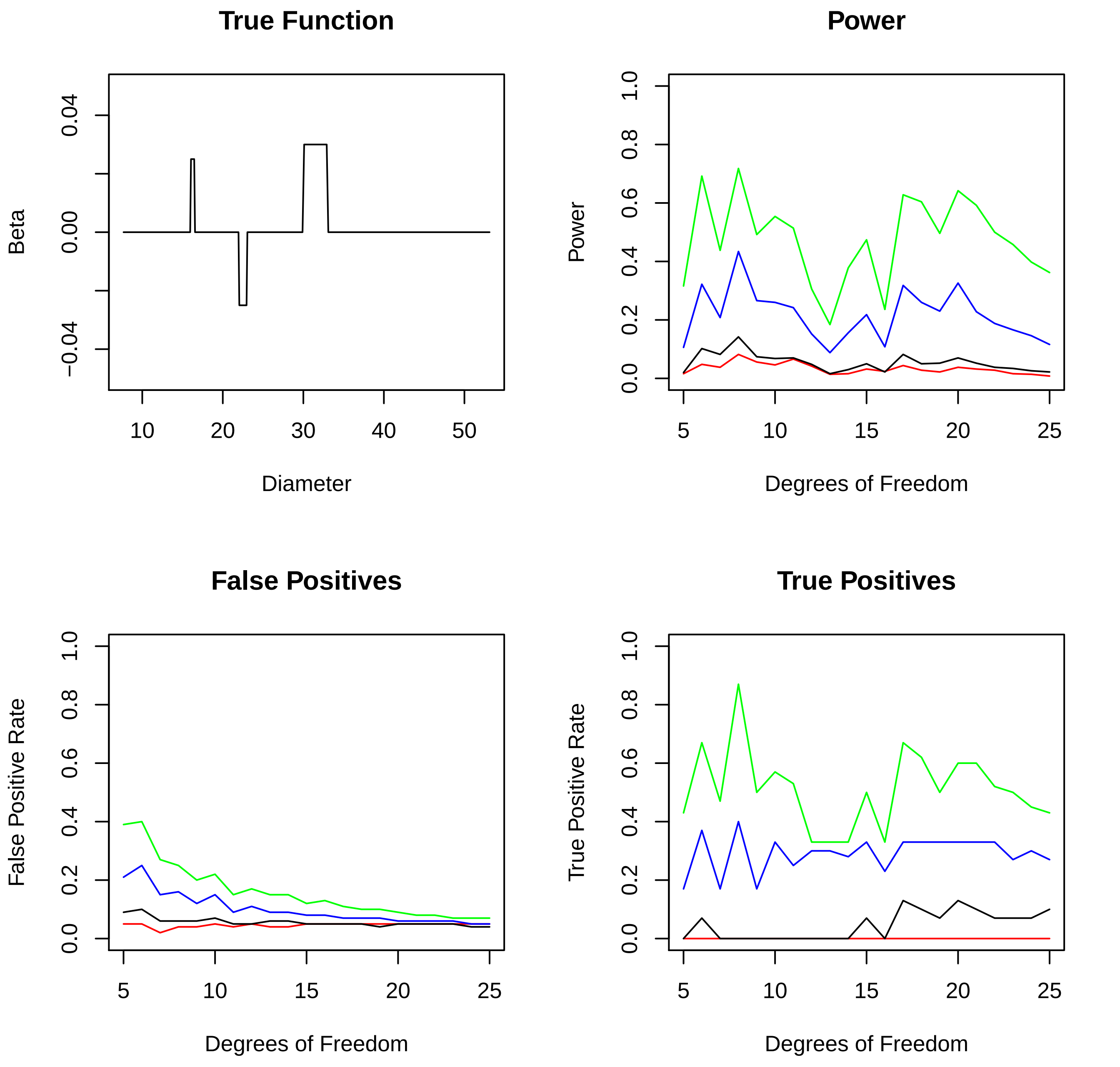
True Function
Beta
0.04
0.00
−0.04
10
20
30
40
50
Diameter
Power
1.0
0.8
0.6
0.4
0.2
0.0
5
10
15
20
25
Degrees of Freedom
False Positives
False Positive Rate
Degrees of Freedom
True Positives
True Positive Rate
Degrees of Freedom

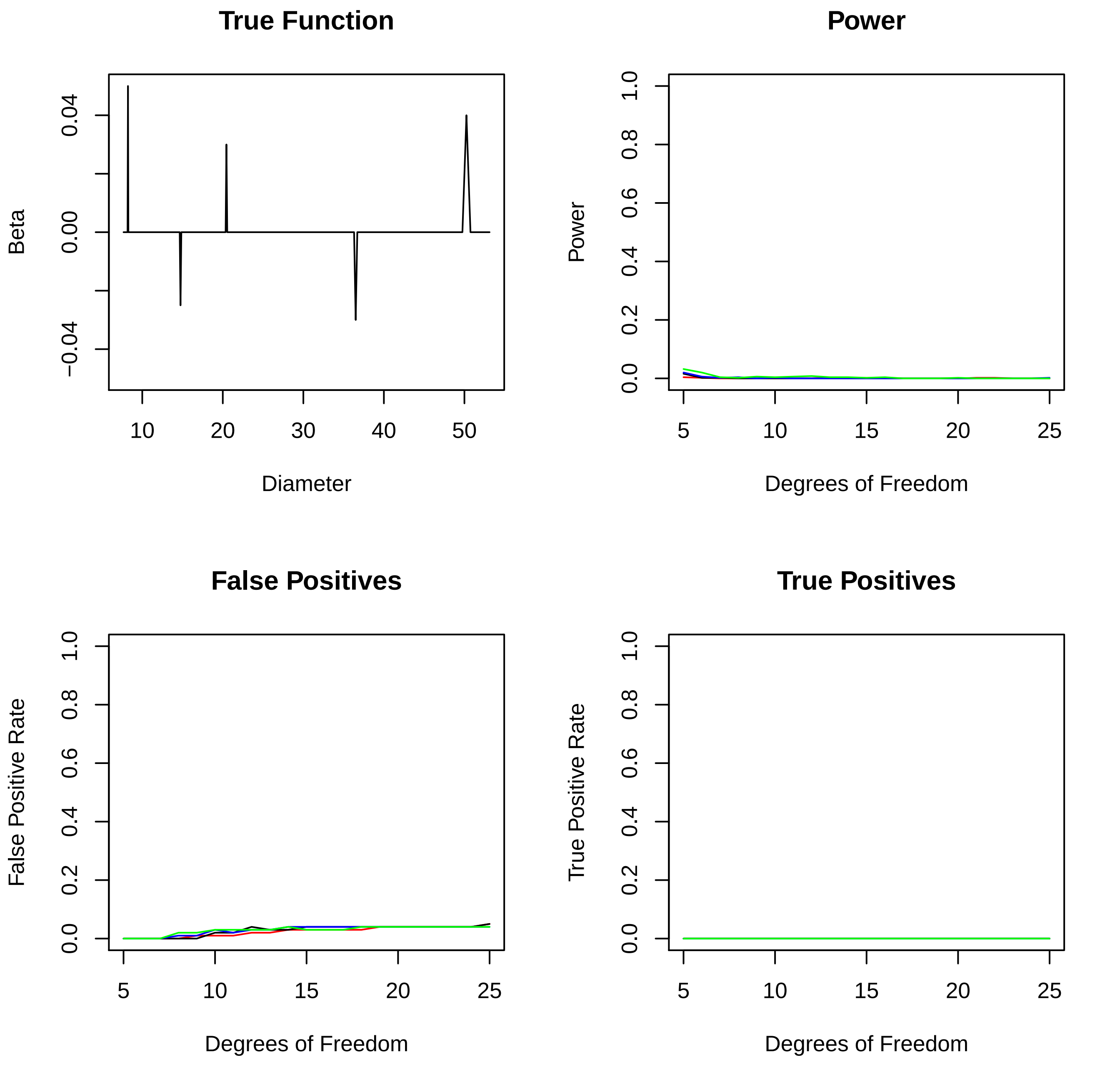
True Function
Beta
0.04
0.00
−0.04
10
20
30
40
50
Diameter
Power
Power
1.0
0.8
0.6
0.4
0.2
0.0
5
10
15
20
25
Degrees of Freedom
False Positives
False Positive Rate
1.0
0.8
0.6
0.4
0.2
0.0
5
10
15
20
25
Degrees of Freedom
True Positives
True Positive Rate
1.0
0.8
0.6
0.4
0.2
0.0
5
10
15
20
25
Degrees of Freedom
